# Supplementary material for: Are carbon emissions trading and green financial instruments synergistic? -Comprehensive quantitative research based on content analysis
Source: PLoS One. 2024 Mar 7;19(3):e0298601. doi: 10.1371/journal.pone.0298601 (PMC10919601; doi:10.1371/journal.pone.0298601)
Supplement: S1 Data — (ZIP) [file pone.0298601.s001.zip › Data/地级市Z_F工作报告.zip!/╣π╢1⁄2/╓╨╔╜╩╨/└┤╫╘╣1⁄2╓┌║┼í╛┬φ┐╦╩2╛▌═°í┐_2010.docx]

2009年工作回顾

　　2009年是全市克难奋进，夺取保增长促发展重要成绩的一年。面对国际金融危机的严峻形势，面对实施《珠江三角洲地区改革发展规划纲要》（简称《规划纲要》）的历史机遇，面对落实“三促进一保持”的重要任务，市政府在省委、省政府和市委的正确领导下，在市人大及其常委会和市政协的监督支持下，坚持用科学发展观总揽全局，全力以赴保增长，多管齐下扩内需，抓住时机促转型，千方百计惠民生，实现了经济社会平稳较快发展。

　　保增长促发展成效显著。把保增长作为经济工作重心，主要经济指标稳健上升。全市实现生产总值1564亿元，增长10.2%。全社会工业总产值4439亿元，增长11.2%。服务业增加值616亿元，增长12.5%。地方一般预算收入110.4亿元，增长10.3%。各项存款余额2211亿元，新增贷款增长203%。全社会固定资产投资545.6亿元，增长16.5%。社会消费品零售总额554.8亿元，增长16.3%。发展质量不断提高，单位生产总值能耗下降3.9%，二氧化硫和化学需氧量排放量分别下降5.9%和27.4%。规模以上工业企业经济效益综合指数153.2%，提高8.7个百分点。

产业结构调整优化升级。把调结构作为危中求进的关键，产业结构水平进一步提升。市扶持产业发展资金增至3亿元，三次产业比例为2.8：57.8：39.4。规模以上支柱产业产值增长18.3%，新能源、新光源、健康医药等新兴产业快速成长。先进制造业发展迅速，装备制造业产值1068亿元，增长20.8%。大企业大项目建设加快，骨干企业规模实力明显增强，产值超50亿元企业增至5家。产业集群升级创新试点工作积极推进，建有省级产业集群升级示范区8个。服务业规模不断扩大，服务业增加值对经济增长贡献率达45.6%。服务业工业用水、污水和生活垃圾处理“三费”实现同价。评出中山市服务业十大品牌，加快建设服务业集聚区，中山保税物流中心通过国家验收。金融服务业快速发展，引进3家金融机构，成立4家小额贷款公司，小榄村镇银行开业一年贷款余额6.28亿元，3家企业申报上市获受理。内外源经济协调发展，规模以上民营工业产值1404.8亿元，增长22.2%。进出口贸易总值244.7亿美元，出口总值177.4亿美元，降幅分别由年初26.5%、21.5%收窄到5.6%、5.2%，高新技术产品出口增长23%。实际利用外资7.66亿美元，增长2.8%。加工贸易转内销额增长2.4倍。现代农业加快发展，实现农业总产值75.7亿元，增长3%。

　　实施《规划纲要》开局良好。把“科学发展，先行先试”作为新一轮发展的契机，准确把握我市发展目标和战略定位。按照立足当前、着眼长远、强化协作、彰显特色的原则，积极打造“一个枢纽、两个基地、三个试点”。签署《珠中江区域紧密合作框架协议》和19个专项合作协议，建立联席会议制度。推动三市机动车年票互认和班车公交化进程，深中通道进入工程可行性研究阶段。实施产业和劳动力“双转移”，安置粤东西北地区农村劳动力5.4万人，省级产业转移示范园区建设再上新台阶。

　　投资消费拉动作用明显。把国家利好政策作为支撑，全面促投资扩内需增后劲。更新改造投资增长51.9%，基本建设投资增长23.8%。建设重点项目88个，完成投资95.9亿元，为年度计划105.6%，获得中央和省专项资金3.7亿元。积极开拓国内外市场，举办11个市级会展，在阿姆斯特丹设立中山货常年展览会，组织越南、印度、澳大利亚、北京、川渝、湘桂等经贸活动，签订各类合同额超600亿元。财政补贴家电和汽车下乡2758万元，拉动消费3.2亿元。实施国民旅游休闲计划，旅游收入突破110亿元，增长13.4%。住宿餐饮零售额增长25.6%，商品房销售额增长83.5%。嘉明电厂二期扩建、500千伏桂山输变电工程、北部组团垃圾综合处理基地一期、中顺大围达标加固、西河水闸重建、保税物流中心、市家电创新中心、纪念中学扩建、市一中初中部迁建、职业技术学院三期、技师学院黄圃校区一期、泰安市场改造、廉租解困房等项目投入使用。新岐江公路、黄圃和三角热电联产、中山中专二期、南部组团垃圾综合处理基地等项目加快推进。

　　自主创新能力不断增强。把创新作为提升经济核心竞争力的基础，积极推进十大科技创新工程。新增省级高新技术企业63家、省级以上工程中心和技术中心16家，获省科技进步奖11项，成为全省首批科技金融结合试点城市。风力发电、半导体照明、电动汽车动力装置等关键技术取得突破，高新技术产品产值占工业总产值30.5%。知识产权、技术标准、名牌带动战略成效明显，发明专利授权量增长108.2%，国家火炬计划日用电器特色产业基地、省新光源高新技术应用基地、广东健康医疗信息技术服务区、省质量监督红木家具和办公家具检验站落户我市，新增中国驰名商标5个、省级名牌名标49个。产学研合作不断深化，建成省部产学研创新联盟2个、示范基地9个，省部企业科技特派员230名专家入驻我市。

　　城乡统筹发展深入推进。把协调发展作为工作重点，加快城乡一体化建设。城镇居民年人均可支配收入23088元，增长7.1%；农民人均年纯收入12288元，增长11%。全市财政转移支付1.2亿元，完成南朗、横栏、三角、黄圃镇一级财政管理，四镇工业总产值、国地两税收入分别比2007年增长43.2%和49.2%。87%村完成村级统一核算改革，35个经济欠发达村全部实现集体年纯收入超100万元。农村土地适度规模经营步伐加快，流转面积46.7万亩。完成农路硬底化200公里，实现1.2万亩农地连片开发，建设现代标准农田8000亩，建有现代农业园区4万亩。完成各镇控规和村庄规划编制任务，建成63个农村居民集中居住小区。投入公共交通补贴3100万元，实现公交班车“镇镇有站、村村有亭”，获得中国人居环境范例奖。生态环保建设取得新成绩，顺利通过全国生态市验收，总量减排和珠江综合整治考核全省第一。18个镇污水处理厂首期全部通过环保验收，全市建成配套管网750公里，污水集中处理能力达86%，中心城区生活垃圾无害化处理率达100%。提前一年完成省政府下达的城乡水利防灾减灾工程任务。山地林相改造5400亩，林木绿化率达25.7%。

　　社会民生事业全面进步。把民生放在发展的突出位置，人民生活质量不断提高。市财政支出59%用于公共服务事业。安排2500万元和提取失业保险金推进就业创业，帮扶困难企业稳定就业岗位，新增就业10.6万人，培训6.8万人，高校毕业生就业率为96.9%，大嫂工作坊、社区车间和农业工作坊等帮助1万多人就地就业，城镇登记失业率为2.16%。城镇社会保险总参保475万人次。城乡居民参加基本养老保险68.5万人，实现自愿参保农民全覆盖。在城乡居民住院基本医疗保险全覆盖基础上，把本市高校和中等职业学校学生纳入参保范围。参加门诊基本医疗保险87.9万人，覆盖率达97.7%。全市财政投入29亿元发展教育事业，中小学优质学位达95%以上，高等教育毛入学率达52.7%，高考录取率连续四年全省第一，民办教育等级学校比例提高到61.4%,各级各类教育均衡协调发展。推行中小学教师工资待遇“两相当”，妥善解决代课教师问题。公共卫生服务日益完善，优化社区卫生站服务功能，加强传染病防控。城市文化实力不断提高，举办首届“中山杯”华侨文学奖、首届中国农民合唱大会，完成国家历史文化名城申报。公共文化体育设施建设加快，做到镇镇有图书馆和健身广场、村村有农家书屋和健身苑。群众体育蓬勃发展，第二届省体育大会金牌总数和团体总分双列全省第三，成功举办第六届市运会。实行人口计生层级动态管理责任制，人口自然增长率为4.89‰。探索农民工进城制度设计，推行流动人员积分制管理。深入开展“五五”普法和法律援助工作，建设基层综治信访维稳中心。平安中山建设加快推进，连续第四次被评为全国社会治安综合治理优秀地市。加强安全生产和食品医药、消防交通安全管理，事故宗数和经济损失分别下降36.9%、28.9%，做好城市管理行政执法工作。市财政用于最低生活保障和社会救济2615万元。对口援建汶川县漩口镇88个项目基本完成，实现中央部署“三年任务两年基本完成”目标。蝉联全国文明城市称号，以和谐社会环境庆祝新中国成立60周年。

　　公共服务能力不断加强。把改革作为政府自身建设的动力，提升科学管理效能。开展政府机构改革。在全省率先成立行政复议委员会，规范行政处罚自由裁量权。以取消市级审批事项和完善“一网式”审批为抓手，推进创新审批方式改革试点工作。依法取消或停止一批行政收费，减轻社会负担1.25亿元。完善激励型财政分配体制,开展财政专项资金绩效管理。压缩行政办公费用，暂停公务用车购置和办公设备更新，一般性行政经费支出降低16%。市属企业创新投融资模式，改革用人薪酬制度，增强了运营活力。深化政务公开，完善电子监察系统建设，廉政勤政和反腐败工作不断加强。坚持科学民主依法决策，办理人大代表议案1件、建议105件，政协提案217件，答复率100%。外事侨务港澳事务服务发展贡献明显，民族宗教工作促进和谐成效突出，商会行业协会等社会组织作用增强。做好统计审计工作，双拥和国防建设不断加强，妇女儿童、老龄、残疾人、慈善事业、气象、人防、档案、方志等各项工作取得新成绩。

　　各位代表，回顾过去一年工作，我们深刻体会到，全市人民同心协力，广大企业同舟共济，我市经受住了历史罕见的国际金融危机重大考验，保增长促发展一系列措施得力有效。一是政策带动。坚持把政策扶持作为促进经济发展的重要手段，做到见事早、行动快、措施准、工作实。及时出台扶持中小企业融资、鼓励进出口、推动固定资产投资、补贴企业参展、鼓励企业创业板上市、促进就业创业等一揽子经济政策，提振社会信心，遏止经济下滑势头，有力带动了经济稳健增长。二是投资拉动。坚持把扩大投资作为保增长强后劲的重要抓手，发挥财政杠杆作用，引导社会资金和金融资本进入投资领域，引导产业资源和科技力量投入结构调整，使应对危机成为打基础、促转变、上水平的过程，有力拉动了经济加快转型。三是市场驱动。坚持把市场开拓作为扩内需稳外贸的重要途径，实施内外市场两手抓，积极开展国内外经贸活动，有力驱动了内外源经济发展。四是服务推动。坚持把优化行政服务作为帮助企业的重要举措，及时召开政府月度经济分析会和双月度重点项目推进会，加强经济运行监测和科学调度，派出基层经济工作服务队，有力推动了企业渡难关谋发展。五是民生优先。坚持以人为本，越是困难时候越是高度重视民生，努力保障市场供应和物价稳定，加快城乡基本公共服务均等化，让全体人民共享改革发展成果，有力营造了社会和谐环境。

各位代表，过去一年的成绩确实来之不易，取得这些成绩，是省委、省政府和市委正确领导的结果，是全市人民艰苦奋斗的结果，也是各位人大代表、政协委员监督支持的结果。我谨代表市人民政府，向全市广大工人、农民、知识分子、干部职工、驻我市人民解放军指战员、武警官兵、人民警察和各界人士表示崇高敬意！向长期关心支持参与中山建设的港澳台同胞、海外侨胞和国内外友人表示衷心感谢！

在充分肯定成绩的同时，我们清醒认识到，我市经济社会发展还存在一些突出问题，主要是：经济回升基础还不稳固，产业结构调整任务紧迫；民间投资有待拉动，外贸形势依然严峻；发展后劲亟需增强，环境代价和社会成本仍然偏高；健康、安全、稳定等涉及群众切身利益的工作还有薄弱环节，政府公共服务有待进一步完善等。对此，我们必须采取有力措施予以解决。

2010年工作安排

　　2010年是我市巩固经济企稳向好态势、完成“十一五”规划目标、全面加快科学发展的关键一年，做好今年工作意义更加重大。纵观国内外形势，今年将总体趋好。国际经济开始复苏，我国经济进入上升通道，加快发展的积极因素增多。但国际金融危机影响还将持续，经济发展的可变因素仍然较多，增长的稳定性面临考验。国内外形势变化对我市经济建设影响明显，但我们在多年发展尤其是去年以来应对国际金融危机的实践中，锻炼了队伍、站稳了脚跟，积累了经验、增强了实力，为抓机遇、迎挑战、促发展奠定了坚实基础。在市委领导下，我们有信心有能力，与全市人民一道，下定决心、真抓实干，破解难题、开拓奋进。市政府努力做到帮扶企业发展的力度不改变，促发展增后劲的投入不降低，人民群众得到的实惠不减少，勤俭节约办实事的作风不动摇，促进经济总量与人均指标、发展质量、人民幸福感的全面进步。

　　2010年政府工作的总体要求是：深入落实科学发展观，全面贯彻党的十七大、省委十届六次全会和市委十二届五次全会精神，积极实施《规划纲要》，以调结构、增后劲、促发展、惠民生为重点，坚持走内涵提升为主的道路，转变发展方式；坚持先进制造业与现代服务业双轮驱动，调整经济结构；坚持自主创新与体制创新，增强发展动力；坚持统筹城乡与生态文明建设，促进社会和谐，取得“三个适宜”新型城市建设更大成就。

　　2010年经济社会发展的主要预期目标是：生产总值增长10%，单位生产总值能耗下降3.9%，二氧化硫和化学需氧量排放量分别下降5.4%和38.8%，全社会固定资产投资增长16%，社会消费品零售总额增长14%，外贸出口总值有所增长，地方一般预算收入增长8%，城镇居民年人均可支配收入增长6%，农民人均年纯收入增长8%，居民消费价格总指数升幅在3%以内，城镇登记失业率控制在3.5%以内，人口自然增长率控制在7‰以下。

　　为实现上述目标，必须做好以下八方面工作。

　　一、拓宽经济增长空间，提高持续发展水平

　　深入贯彻《规划纲要》。着眼珠三角和我市发展大局，努力建设珠三角三大经济圈的重要交通枢纽，建设沿海产业带先进制造业基地、珠江口西岸重要服务业基地，建设全国统筹城乡发展综合改革试点、全国创新审批方式改革试点和广东省产业集群升级创新试点。安排产业发展专项资金3.2亿元，加大产业升级创新扶持力度。加强专项规划对接，提升中山区位新优势。完成“十一五”规划目标任务，科学编制“十二五”规划。

　　增强内需拉动作用。安排市重点项目116项，其中年度项目92项，年度计划投资185亿元，保持投资规模合理较快增长。引导资金加大向基础设施、结构升级、技术改造、新兴产业、低碳经济、民生保障的投入力度。鼓励固定资产投资，用足各级重点项目扶持政策，积极争取中央和省等各类资金。探索激活民间投资创新机制，出台鼓励社会投资政策，降低市场准入门槛，支持民间资本进入公共服务领域。提高城乡居民特别是低收入群体的增收水平和消费能力，培育新的消费增长点。用好国家引导消费政策，提升居民消费信心。发展消费信贷，促进家电、汽车、住房、旅游、文化、信息等消费。控制房价过快增长，保持房地产业稳健发展。完善农村流通网络，发展农村消费市场。

　　积极发展地方金融。创建城乡金融服务一体化综合改革试点，做好科技金融结合试点工作，推动金融业改革创新发展。完善金融服务体系，引进培育各类金融机构，支持境内外金融机构在我市设立区域总部和后援基地。开展“政银企”合作，完善中小企业信用担保体系，创新金融产品。推动农信社改制为农村商业银行，发展村镇银行、小额贷款公司和融资性担保公司，完善政策性农业保险，鼓励企业上市发展。防范化解金融风险，优化金融生态环境。

　　加快推进“三旧”改造。科学用好城市发展资源，编制旧城镇、旧厂房、旧村庄改造规划，出台“三旧”改造实施意见，年内完成1万亩“三旧”改造。以石岐区、小榄镇和南头镇作为市镇（区）共建改造试点，发展创意产业、创业孵化、文化产业、商贸物流、生产服务业，确保全市“三旧”改造一年初见成效、两年突破性进展。加强土地资源整合开发，回收利用闲置土地，做好园地山坡地补充耕地工作，节约集约用地。盘活国有资源，制定市属国有经济发展规划，优化重组市属国有企业。

　　提高自主创新能力。完善以增强经济核心竞争力为目的、企业为主体、市场为导向、产学研相结合的技术创新体系，市财政科技投入2.8亿元。扶持建设工程技术中心、科技服务共性平台、产业集群创新联盟，重点建设好半导体照明产业技术联盟、市家电创新中心，支持国内外重点院校和科研机构服务中山科技创新。围绕产业集群升级和新兴产业发展，开展示范工程和科技强企支撑计划，培育科技型龙头企业。深化品牌建设、技术标准和知识产权战略，落实“千名万人”科技人才行动计划。

二、加快产业结构升级，提高经济效益水平

大力发展新兴产业。制定战略性新兴产业发展规划和扶持政策，重点发展风电装备、太阳能设备、新型高能电池、电动汽车动力装置等新能源产业，半导体照明等新光源产业，生物医药、中医药、医疗设备等健康产业。推动开发具有自主知识产权的核心装备和关键部件，加快发展高新技术产业。培育低碳经济，发展节能环保和绿色生态产业，建设资源节约型和环境友好型产业环境。

优先发展现代服务业。创新服务业发展机制，完善服务业发展目录，重点发展金融服务、现代物流、信息服务、科技服务、商务会展、服务外包、文化创意和总部经济等。支持服务业为制造业提供研发设计、物流配送和购销渠道，鼓励发展新型业态。加快服务业集聚区和商贸功能区建设，争取省级服务业集聚区认定，支持服务业企业向规模化发展。建设东区中心商务区、西区服务业综合改革试验区、华南家电产业服务创新区、南朗滨海温泉度假城、5A级孙中山故里旅游景区等重点项目。整合优化旅游资源，促进旅游与文化、工业、生态农业融合发展。加强服务业区域合作，引进高端服务业机构。理顺服务业价格政策，实施服务业品牌和标准化战略，加强服务业统计。

积极发展先进制造业。坚持高级化与适度重型化方向，推动制造业升级发展。完善产业园区基础设施，加快建设临港装备制造基地，建成投产10大工业项目。发展船舶和海洋装备、大型铸锻件、新型平板显示、电梯等装备制造企业，争取一批省级以上重点扶持先进制造业项目在中山布局。推进信息化、数字化、智能化与工业化融合，引导企业开展技术研发、产品创新和完善管理，扩规模增效益。鼓励企业通过并购、重组、联合等方式，增强制造业品牌、技术、市场、人才竞争力。培育引进一批业绩突出、带动性强、贡献度高的大企业大项目，加快产业集群升级创新，发展百亿元企业，建设千亿元产业集群。

提升发展传统产业。推动传统产业由低端向高端、由制造向创造转变，下决心运用市场和行政手段，淘汰一批落后产能。加强高新技术、先进适用技术和现代管理在研发设计、原料控制、生产制造、企业管理、品牌建设等环节的应用，拓展电子商务平台，延伸集群产业链条。改造提升精细化工、电子信息、电器机械、五金制品、纺织服装等传统支柱产业。

坚持发展现代农业。构建基础增强、产业优化、装备提升、生态环保、富民增效的农业生产模式，提高农业市场化专业化规模化水平。打造“一带两区十基地”等现代农业园区和标准化示范基地，重点建设国家级现代农业园区南方绿博园。创建万亩现代渔业示范基地，推行健康养殖。加强农业信息化建设，发展农产品专业市场和加工物流配送企业。争创国家和省级农业名牌产品，组织申报无公害农产品、绿色食品资格认证。强化农产品标识管理和质量安全监测，做好动植物疫病防控工作。稳定粮食生产，确保粮食安全。

三、积极发展区域合作，提高经济开放水平

加快推进区域合作。立足珠中江，承接广佛肇、深莞惠，融入大珠三角城市群，提高区域合作水平。落实珠中江区域紧密合作框架协议，编制执行专项规划。积极参与珠中江基础设施、产业布局、城市规划、环保生态、公共服务“五个一体化”，让人民群众共享区域合作成果。有序推进产业和劳动力“双转移”，建设好省级产业转移示范园区。全面完成汶川县漩口镇对口援建任务，做好汕尾市80个贫困村“规划到户、责任到人”扶贫开发工作。

拓展内外两个市场。鼓励企业建立连锁经营、销售网络和流通体系，推动中山产品“北上西进”，加快发展国内市场。办好13个专业会展，做好国内外采购团组织工作。帮助企业走出去找市场，搭建“中山货”出口促销平台，建立国外营销网络。稳定香港、欧美、日本等主要出口市场，开拓东盟、南美、中东等新兴市场，加强与澳大利亚、印度、非洲等经贸合作。抢抓出口订单，增加出口货源，壮大出口增长点。扩大先进技术装备和资源性原材料进口。完善外经贸政策服务体系，鼓励企业运用出口信用保险化解收汇风险。积极应对国际贸易壁垒和贸易摩擦，支持企业利用国际通行规则维护权益。力促对外贸易稳定增长。

转变外贸增长方式。提升出口商品质量档次、品牌形象和增值率，扩大机电产品和高技术产品出口规模效益。发展服务贸易和技术贸易，促进外贸结构升级。鼓励符合政策的来料加工企业不停产转为“三资企业”或民营企业，发展加工贸易深加工结转业务。推动加工贸易由外销向内外销结合转型，由单一加工制造功能向研发设计、品牌营销领域延伸，由外资企业为主向内外资并举发展。充分发挥中山保税物流中心作用，推行属地申报的口岸验放新型模式，优化通关环境。

加大招商引资力度。鼓励社会化专业化市场化招商，提高利用外资水平。吸引世界500强企业、国际大财团到我市设立产业项目、地区总部和研发机构，促进外商投资产业链向研发设计、核心部件生产和物流配送环节伸展，扩大服务业吸收外资成效。通过股权招商等模式，引导跨国公司和国际资本参与民营企业改组改造。大力吸引内资，重点引进央属省属和优质民营企业项目。

四、全面构建宜居中山，提高城市建设水平

科学谋划宜居城乡建设。打造生产发展、生活富裕、生态良好、文化繁荣、社会和谐、人民群众具有幸福感的宜居城乡。坚持改善人居环境，实现城乡空气质量达二级天数占全年90%以上。优化水资源配置，完善供水安全保障体系，着力解决咸潮期间用水需求。发展适合农村特点的清洁能源。继续实施绿化中山大地工程，积极推动“绿道网”中山段规划建设，强化生态公益林保护，提升城乡绿化净化美化水平。

加快交通基础设施建设。构建“全域中山”公路网络，加快改善与周边城市的交通对接。年内实现新岐江公路、中山三路下穿隧道、105国道沙朗古鹤段改造、十水线古镇段、康华路改造等项目投入使用。续建福源路二标、东部快线东部特大桥、阜港公路改造等项目。新开工建设广珠中线二期、长江路、城桂公路和南区中环路改造、东部快线起点及榄横路段、十水线鸡鸦水道和小榄水道特大桥等项目。推进深中通道、小榄快线、古神公路二期、加八线、横四线西段、横二线东段、黄圃岭栏路等项目前期工作。积极参与西部沿海铁路、广佛江珠城际轻轨、广珠西线高速公路三期建设，加快与珠三角区域公路、铁路、港口、机场、轨道交通等无缝对接。坚持“公交优先”和全市公交“一盘棋”，加强建成道路养护管理，加快公交站场枢纽、首末站、候车亭建设，构建畅通便捷的公共交通网络，提高公交出行分担率。

完善城市功能配套建设。优化城市承载功能，年内完成市实验高中和市一中高中扩建、第一中等职业技术学校、新中医院、人民医院杨郭恩慈大楼、新社会福利院、中心粮库二期等项目。加快建设黄圃港区、黄圃和三角热电联产、220千伏胜龙等3个输变电工程、石油化工储备区、天然气利用等项目。新开工建设档案信息中心、第二人民医院二期、第三人民医院扩建等项目。推进新图书馆、湿地生态公园等项目前期工作。

深入开展生态文明建设。巩固全国生态市建设成果，创建全国生态文明建设试点，市财政环保投入增长28%。做好总量控制、污染减排工作，严格执行节能减排问责制和“一票否决”制。实行建筑节能、节约用水、循环利用、清洁生产，落实节能减排评估考核制度。开展全民节能行动，推广绿色消费模式。力争8个镇污水处理厂二期扩建、固体废物处理中心年内投入运行，推进南部组团垃圾综合处理基地、镇区生活污水处理配套管网建设。完善污染源长效监管机制，完成大气环境、江河水质和机动车尾气在线监控系统建设。

五、深化城乡体制改革，提高统筹发展水平

加快统筹城乡综合改革试点建设。编制统筹城乡发展战略规划，完善城乡交通、能源、信息、教育、卫生、文化等公共基础设施，新增城市维护费向农村基础设施建设维护倾斜。推动统筹城乡示范镇区建设，加快城中村改造和村改居工作，建设城乡新社区。实施政策性农村住房保险，统筹规划建设农民居住小区。按照省统一部署，做好强镇扩权工作，强化镇区面向群众的社会管理、公共服务和市场监管职责。以城镇化加快城乡一体化，城镇化率达到87%，提高城市化建设水平。

加强城乡公共财政体制改革。扩大公共财政服务范围，推进基本公共服务均等化。市级财力61%用于公共服务，新增财力主要投向社会公共事业、基础设施建设和扶持企业发展。坚持科学依法治税理财。对民众、阜沙、板芙、神湾、大涌五个镇实施两年一级财政管理，给予“一级财政＋专项资金补贴+固化债务”政策，推动区域协调发展。深化财政支出绩效管理改革，完善专项资金分配管理办法，提高资金分配透明度和规范化水平。开展财务核算集中监管试点和公务卡结算试点改革，加强政府采购。建设全省价格改革试点市。合理有偿使用国有公共资源，规范非税收入管理。坚持开源节流，提高财政资金放大效益。

深化农村体制改革。年内基本完成村级统一核算，完善农村集体经济发展机制。加强土地承包经营权流转管理服务，构建市镇村三级流转服务平台。倡导村级集体经济组织以股份合作集中流转土地，采取多种流转形式推动土地适度规模经营。促进农村集体经济与社会事务分离，探索农村集体经济运营新方式，严格落实村务财务公开民主管理。稳妥推进集体林业产权制度改革。

完善农村基础设施。市财政投入3亿元加强农村基础设施建设。健全农村公路客运系统，实现镇通行政村公路路面硬底化。启动新一轮水利防灾减灾工程，加快防洪排涝设施建设，提高城乡排涝标准。综合整治内河涌260公里，改善水环境质量。建设硬底化主干农路200公里，打造3.5万亩未通路桥农田路网，完成78座危桥改造，高标准整治鱼塘4万亩，加强基本农田建设4000亩。创建镇村小康环保生态示范村，营造农村优良生产生活环境。

六、大力发展社会事业，提高城市文明水平

优先发展教育事业。市财政投入10亿元加快教育现代化。推进中小学布局调整、振兴初中行动计划、学校优质化工程、“四名”工程和名校带动战略。促进学前教育规范发展，提高义务教育均衡化优质化水平，以“扩容促优”为主线发展高中阶段教育。加快五桂山职教园区建设，超常规发展职业教育。支持高等院校加强适合中山发展的学科建设，鼓励产学研合作。创新教育融资渠道和办学模式，壮大优质民办学校。推行学校后勤服务社会化。全面落实中小学教师工资待遇“两相当”。坚持人才强市战略，完善人才政策体系，建设技能型和创新型人才队伍。

加强文化名城建设。提升市民素质，夯实文明基础。加快创建国家历史文化名城，做好“文明城市建设提升年”工作。构建覆盖城乡的公共文化服务体系，开展孙中山文化系列活动，筹备纪念辛亥革命100周年工作，做优慈善万人行、中山合唱节等品牌。发掘镇区特色文化和民间艺术资源，加强历史文物保护。加快发展游戏游艺、动漫设计等文化产业，提升产业文化内涵。做好上海世博会参展工作。

发展医疗卫生事业。积极稳妥推进医药卫生体制改革，建立科学合理的医疗卫生机构管理体制和运行机制，引导民间资本举办医疗机构。加快城乡基本医疗卫生均等化，完善农村社区卫生服务体系，逐步形成社区首诊、分级医疗和双向转诊新格局，实现社区卫生服务全覆盖。发展中医药健康服务，全面实施妇幼安康工程，加强传染病防控。构建和谐医患关系，为群众提供安全有效、方便廉价的医疗卫生服务。

推进体育、人口计生等工作。以广州亚运会为契机，广泛开展全民健身运动，做好我市运动员参赛等工作。落实人口计生工作层级动态管理责任，深入开展“镇区无政策外多孩出生、村（社区）无政策外出生”活动，创建全国计划生育优质服务先进单位。完善流动人口服务管理模式，全面实施流动人员积分制管理，继续开展流动务工人员百佳评选。落实优抚对象抚恤补助待遇，提高双拥工作水平。做好外事、侨务、港澳事务、台务、民族、宗教、国防建设、人防、气象、地震、档案、方志等工作。

七、坚持改善民生保障，提高社会管理水平

促进市民就业创业。安排2500万元和提取失业保险金推进就业创业，建设国家级创业型城市。把就业新增长点与经济新增长点相结合，年内新增就业6万人。建立外省市劳务输入基地，保障企业用工需求。解决零就业家庭和困难群体就业问题，推广大嫂工作坊、社区车间和农业工作坊等就业形式。开展农村富余劳动力技能培训，加快农村劳动力转移就业。加强校企合作，扩大就业见习基地建设，促进高校毕业生和新增劳动力就业。完善就业公共服务，建设中心镇区人力资源市场。落实全民创业计划，实施青年创业工程，建设创业孵化基地，促进创业带动就业。开展扩大失业保险基金支出范围试点，建立集保障生活、预防失业、促进就业三位一体的失业保障机制。

加强社会保障服务。市财政投入3.35亿元用于社会保障。推进城乡居民养老保险一体化，将城镇无业居民纳入养老保险制度管理，加快农村养老保险向城镇职工养老保险过渡，逐步提高农保待遇。完善城乡医疗保险体系，实现城乡居民门诊基本医疗保险全覆盖。推进全民安居工程，支持居民自住和改善性购房需求。完成第二期廉租房建设和农村低保家庭危房改造，优化实物配租和租金补贴相结合的住房保障。安排3100万元用于最低生活保障和社会救济，创新慈善超市救助经营模式，逐步提高困难群体保障水平。大力发展社会服务事业，做好老龄、妇女儿童、残疾人等工作。

建设和谐平安中山。完善社会管理工作，加强社会组织、网络虚拟社会等重点领域管理。扩展基层综治信访维稳中心覆盖面，建设适应社会转型的利益协调、诉求表达和权益保障机制，形成“大信访、大调解”格局。推行工资保障金制度，打击恶意欠薪逃匿行为，构建和谐劳动关系。开展市容市貌综合治理，加快问题路段交通改造，用好各类停车资源，整治非法营运、交通违法和房屋违法转功能行为，提高城市管理水平。做好法制宣传和法律援助工作，严厉打击犯罪活动。加强安全生产、消防交通、食品医药等安全工作，遏制重特大事故发生。强化社会治安、公共安全、流行疾病、自然灾害等突发事件的防控处置机制。

八、加强政府自身建设，提高行政服务水平

认真履行政府职能。加强政府公共服务管理，进一步理顺部门职责关系，合理划分决策权限，严格规范决策程序，完善行政问责制度。实行事业单位分类改革，规范机关事业单位人员聘用管理。充分发挥商会行业协会等社会组织作用。探索基本公共服务多样化供给形式，引导社会组织和个人参与公益服务。强化风险管理，健全各级各部门应急管理机制，提高干部应对突发事件能力和应急处置水平。

深化审批制度改革。完善政府公共服务模式，推进创新审批方式改革试点建设。建立行政审批事项目录管理制度，创新直接面向群众的“窗口”机构服务管理，构建辅助“网上审批”有形服务体系。发挥政府门户网站和行政服务热线作用，优化政府与公众互动平台。提高政务公开水平，建立鼓励公众参与、重视专家论证的决策咨询机制。

坚持依法民主行政。依法管理经济社会事务和行使职权，规范行政自由裁量权和行政复议工作，开展法治城市、法治镇区创建活动。自觉接受人大的法律监督、工作监督和政协的民主监督，定期向市人大报告工作和向市政协通报情况，认真落实人大各项决议决定，及时办理人大代表议案建议和政协委员提案。充分听取各民主党派、工商联、无党派人士和各人民团体意见，依法决策、民主决策、科学决策，巩固和扩大基层民主。

建设廉洁高效政府。坚持执政为民，全心全意为群众办好事，真抓实干为企业谋发展。以目标倒逼进度，以督查倒逼落实，推进行政提速和服务增效。坚持艰苦奋斗，勤俭节约，实现行政运行经费“五个零增长”和一般性支出预算压减5-10%。坚持加强学习，建设马克思主义学习型政府，促进公务员知识结构转型升级，增强政府推动科学发展的能力。坚持严守纪律，严格执行廉洁从政各项制度，健全行政监察派驻工作，治理商业贿赂，强化经济责任审计，加强行政监察和行政问责。建设重学习、重调研、重服务、重落实的工作作风，建设为民务实、廉洁高效的公务员队伍，建设人民群众满意的政府。

各位代表！新的一年，我们肩负着更加艰巨而光荣的任务。目标和机遇激励着我们，责任和使命鞭策着我们。人民的智慧，是我们科学发展的良方经略；人民的重托，是我们一往无前的力量源泉；人民的利益，是我们一切工作的起点和归宿。让我们同心同德、锐意进取，在建设“三个适宜”新型城市的道路上，谱写壮丽的篇章，开创美好的未来！
